# Pseudoprogression in Glioblastoma: Role of Metabolic and Functional MRI-Systematic Review

**DOI:** 10.3390/biomedicines10020285

**Published:** 2022-01-26

**Authors:** Ingrid Sidibe, Fatima Tensaouti, Margaux Roques, Elizabeth Cohen-Jonathan-Moyal, Anne Laprie

**Affiliations:** 1 Radiation Oncology Department, Claudius Regaud Institute, Toulouse University Cancer Institute Oncopole, 31100 Toulouse, France; sidibingrid@gmail.com (I.S.); tensaouti.fatima@iuct-oncopole.fr (F.T.); moyal.elizabeth@iuct-oncopole.fr (E.C.-J.-M.); 2 Toulouse NeuroImaging Center (ToNIC), University of Toulouse Paul Sabatier INSERM, 31100 Toulouse, France; margaux.roques@inserm.fr; 3 Radiology Department, Purpan University Hospital, 31300 Toulouse, France; 4 INSERM UMR.1037-Cancer Research Center of Toulouse (CRCT)/University Paul Sabatier Toulouse III, 31100 Toulouse, France

**Keywords:** glioblastoma, pseudoprogression, true progression, MRI, radiomics, MR spectroscopy, artificial intelligence

## Abstract

Background: Glioblastoma is the most frequent malignant primitive brain tumor in adults. The treatment includes surgery, radiotherapy, and chemotherapy. During follow-up, combined chemoradiotherapy can induce treatment-related changes mimicking tumor progression on medical imaging, such as pseudoprogression (PsP). Differentiating PsP from true progression (TP) remains a challenge for radiologists and oncologists, who need to promptly start a second-line treatment in the case of TP. Advanced magnetic resonance imaging (MRI) techniques such as diffusion-weighted imaging, perfusion MRI, and proton magnetic resonance spectroscopic imaging are more efficient than conventional MRI in differentiating PsP from TP. None of these techniques are fully effective, but current advances in computer science and the advent of artificial intelligence are opening up new possibilities in the imaging field with radiomics (i.e., extraction of a large number of quantitative MRI features describing tumor density, texture, and geometry). These features are used to build predictive models for diagnosis, prognosis, and therapeutic response. Method: Out of 7350 records for MR spectroscopy, GBM, glioma, recurrence, diffusion, perfusion, pseudoprogression, radiomics, and advanced imaging, we screened 574 papers. A total of 228 were eligible, and we analyzed 72 of them, in order to establish the role of each imaging modality and the usefulness and limitations of radiomics analysis.

## 1. Introduction

Glioblastoma (GBM) is the most aggressive and frequent type of primary brain tumor in adults [1], with an incidence of about 4–5/100,000 [2]. Median age at diagnosis is 64 years, and the 5-year relative survival rate is about 5% [3,4]. Standard treatment includes surgery or biopsy followed by radiotherapy (RT) combined with chemotherapy using temozolomide [5]. Heterogeneity, infiltration pattern, angiogenesis, and hypoxia, together with their impact on tumor metabolism and radioresistance, are responsible for the two main issues with these tumors. First, the short-lived effect of classic therapeutic approaches, and secondly the need for multimodal imaging to characterize the tumor initially and, in the event of post-treatment changes, to decipher its behavior and propose second line treatments if progression is confirmed [6,7].

Magnetic resonance imaging (MRI) is the best imaging modality for the diagnosis and follow-up of GBM, in a meta-analysis of more than 2000 patients after first-line treatment, 36% of patients (range 3–50% depending on the study) were found to have increased contrast enhancement on their first post-radiation MRI that was not true progression (TP) [8,9,10,11]. Combined radiotherapy and chemotherapy can induce an increase in contrast-enhanced lesions that mimics tumor progression but subsequently stabilizes or decreases without any additional treatment. This pseudoprogression (PsP) is a potential surrogate marker of treatment efficiency. In a prospective study of 463 patients, Wick et al. [12] demonstrated that the incidence of PsP is about 9.3%. They used clinical symptoms to diagnose progressive disease and a new MRI 8 weeks after treatment to diagnose PsP. In a review, differences in the rate of incidence of PsP after RT/temozolomide were mainly explained by the criteria (stringent or liberal) used to diagnose PsP (12% when using stringent criteria, and 23% when using liberal criteria) [13]. Response Assessment in Neuro-Oncology (RANO) criteria are widely used to classify patients according to progressive or nonprogressive disease [14]. However, these criteria, have limitations. As they are based solely on morphological MRI assessment, less than 12 weeks after completion of chemoradiotherapy, tumor progression can only be diagnosed if enhancing lesions appear outside the radiation field. Recent guidelines on the role of imaging in the management of progressive GBM in adults recommend the use of advanced MRI techniques such as diffusion weighted imaging (DWI), proton magnetic resonance spectroscopic imaging, and perfusion weighted imaging to differentiate between PSP and TP [15]. In the present review, we focused on the added value of different types of advanced medical imaging when it comes to differentiating PsP from TP, as well as the role of radiomics in meeting this clinical challenge.

## 2. Materials and Methods

**Figure 1:**
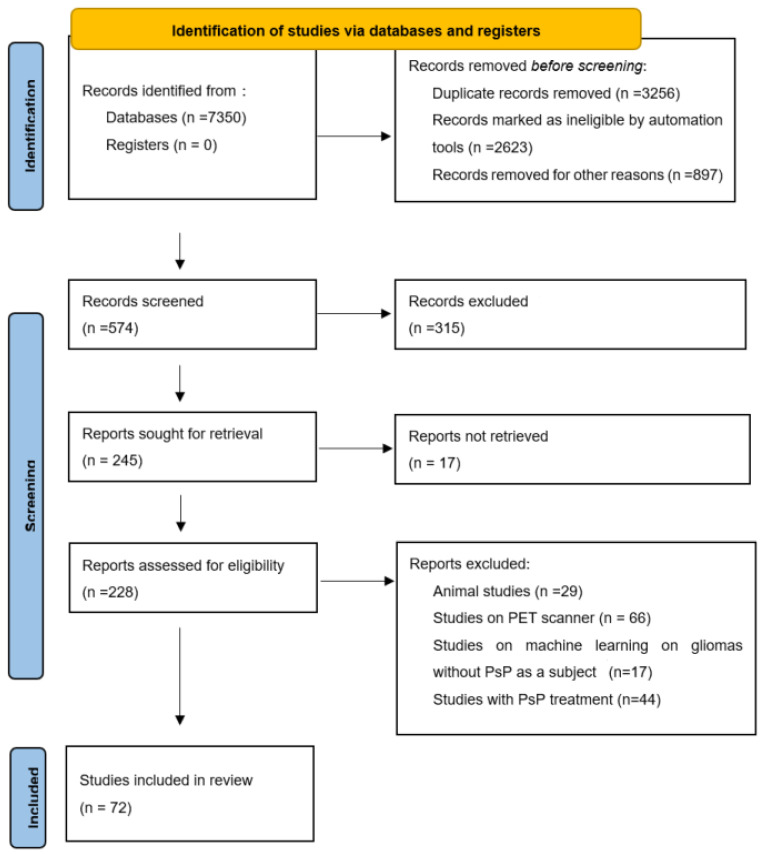
Study flow chart from PRISMA flow diagram 2020 statement. PsP = pseudoprogression, PET = positron emission tomography, PRISMA = preferred reporting items for systematic reviews and meta-analyses.

We conducted a comprehensive search of the PubMed and Google Scholar databases to find relevant articles (published up to September 2021). The search terms were as follows: magnetic resonance spectroscopy or MR spectroscopy or MRS, GBM, glioma, recurrence, diffusion, perfusion, pseudoprogression, radiomics, and advanced imaging. Articles concerning PsP in adult patients with glioma, high-grade glioma, or GBM were examined. References provided by relevant articles were also examined to identify additional studies for inclusion. Articles describing positron emission tomography imaging or animal studies or PsP treatment or machine learning on glioma without PsP as subject were excluded. A total of 228 articles and after applying exclusion criteria 72 were included. Figure 1 shows our study flow chart.

Two persons checked the data: one extracted data and an other person checked the extracted data.

## 3. Results

### 3.1. Definition of Pseudoprogression

PsP can be defined as radiographic changes (enlarged or new contrast enhancement) within the radiation field mimicking TP that spontaneously resolve without any modifying therapy [16]. Clinical definitions of PsP are quite variable, which may explain some of the differences in its reported incidence [16]. PsP mainly occurs during the first 6 months post-RT [8,17], but most studies describe it as occurring within the first 3 months [16]. By contrast, radionecrosis (RN) is observed more than 6 months post-RT (18–24 months to several years post-treatment on average). RN is a late disease corresponding to white-matter necrosis. PsP and RN do not have the same histopathological and biological mechanisms. RN can occur as a result of chronic inflammation and wall thickening, as well as vessel hyalinization and even collapse of the microvessels surrounding the tumor, owing to reactive telangiectasia [18]. In contrast, the mechanisms of PsP are not well-documented. RT probably induces damage to epithelial cells and local tissue inflammation, resulting in edema and abnormal vessel permeability in which vascular endothelial growth factor signaling is upregulated. This, in turn, may cause an increase in edema seen on T_2_-weighted images and/or new or increased contrast agent enhancement [8]. Brandes et al. [9] showed that PsP is more frequent among patients with O6-methylguanine-DNA methyl transferase (MGMT) methylation (MGMT is a DNA repair enzyme that plays an important role in chemoresistance to alkylating agents). The latter have a better median overall survival rate than those without MGMT methylation 18.2 months versus 12.2 months [19] and up to 46 months versus 19 months [9]. It has been reported that there is a 60% probability of early TP in unmethylated MGMT promoter tumors [19]. PsP may represent an active inflammatory response to the tumor [20]; in other words, an enhanced response. In a study of 130 patients, Kucharczyk et al. [21] used RANO criteria to demonstrate that patients with PSP do not differ significantly from patients with stable disease on overall survival (13 months vs. 12.5 months), although they do differ significantly from patients with TP. In the same study, a comparison of response RANO criteria, MacDonald criteria, or Response Evaluation Criteria in Solid Tumors (RECIST) found that the incidence of PsP varied from 15% (RANO) to 19% (MacDonald) and 23% (RECIST). Wick et al. [12] did not find any signs on conventional MRI to distinguish between PsP and TP; the only sign for TP was subependymal enhancement for with 38.1% sensitivity, 93.3% specificity, and 41.8% negative predictive value. In a meta-analysis, conventional MRI (166 patients) had a pooled sensitivity and specificity of 68% (95%CI [51, 81]) and 77% (95%CI ([45, 93]) [22]. Owing to these limitations, other MRI modalities were studied to evaluate their ability to diagnose PsP. No significant differences in progression-free survival were found between two groups, even in patients with MGMT methylation [12], but these authors assessed PsP rates and TP patterns in a phase III trial of bevacizumab plus radiotherapy/temozolomide for newly diagnosed glioblastoma. However, bevacizumab is also a treatment, for radionecrosis [23], so these results are very different from the standard upfront treatment of GBM, which does not include bevacizumab.

**Figure 2:**
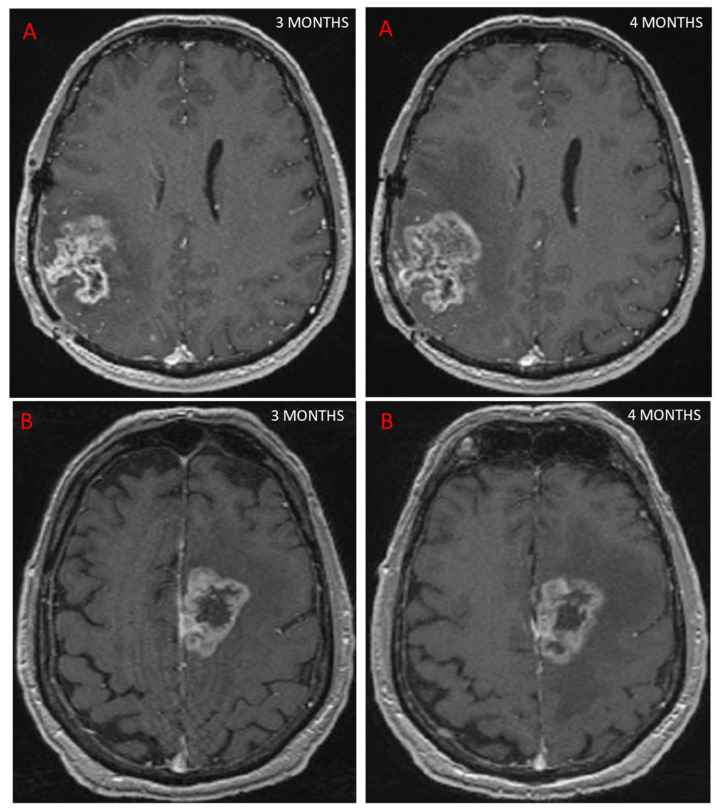
This figure shows two patients treated for GBM with concurrent RT and chemotherapy. At 3 months, MRI showed an increase in contrast-enhancing lesion on axial T1 sequence after injection of a contrast agent, suggestive of progression in both cases. Further MRI scans performed 1 month later (4 months post-RT) showed that one patient had TP, with an increase in contrast (**A**), while the other patient had PsP, as the contrast remained stable (**B**).

Follow-up MRI assessing GBM response to treatment is useful for depicting PsP, as the contrast enhancement portion of the lesion either remains stable or diminishes over time [24]. However, conventional MRI does not allow a reliable distinction to be made between PsP and TP, as both may be characterized by mass effect, perilesional edema, and contrast agent enhancement due to blood–brain barrier breakdown [25], (Figure 2). In a study of 93 patients [25] looking for different signs on conventional MRI to distinguish between PsP and TP, the only sign for TP was subependymal enhancement, with 38.1% sensitivity, 93.3% specificity, and 41.8% negative predictive value. In a meta-analysis, conventional MRI (166 patients) had a pooled sensitivity and specificity of 68% (95%CI [51, 81]) and 77% (95%CI [45, 93]) [22]. Owing to these limitations, other MRI modalities have been studied to evaluate their ability to diagnose PsP.

### 3.2. Advanced MRI and PsP

#### 3.2.1. Diffusion Imaging Including Diffusion Tensor Imaging (DTI) and Diffusion Weighted Imaging (DWI)

DTI provides details on tissue microstructure and organization well beyond the usual image resolution and allowing diffusion anisotropy to be quantified and subtle white-matter changes to be detected [26]. Restricted diffusion due to tumor presence is seen as high-signal intensity on DWI and reduced apparent diffusion coefficient (ADC) values in the solid components of the tumor. Another important element in DWI is the b-value, a factor reflecting the strength and timing of the gradients used to generate DWI. The b-value corresponds to diffusion effects. The ADC is calculated on the basis of the difference in the signal intensity on DW images obtained at two different b values, corresponding to an exponential decrease in signal intensities [27,28].

In a study assessing DWI for its ability to differentiate TP from PsP, ADC values were found to be higher in necrotic tissue than in recurrent tumor tissue [9]. In high-grade gliomas previously treated with standard chemoradiation, the presence of centrally restricted diffusion in a new ring-enhancing lesion may indicate radiation necrosis (RN) rather than tumor recurrence, and this was confirmed by a prospective study of 17 patients with high-grade gliomas who developed a new ring-enhancing necrotic lesion and who underwent re-resection [29]. A comparison of histogram parameters for each ADC map showed that the fifth percentiles of ADC at a b value of 1000 s/mm^2^ (ADC1000) and at a b value of 3000 s/mm^2^ (ADC3000) were significantly lower in the TP group than in the PsP group (*p* = 0.049 and *p* = 0.001). By contrast, the two groups did not differ significantly on either mean ADC1000 or mean ADC3000. The fifth percentile of the cumulative ADC histogram obtained at a high b value (1000 or 3000) in new or enlarged enhancing lesions appears to be a promising parameter for differentiating TP from PsP after GBM treatment, with an accuracy of 88% [30]. Kazda et al. [31] found that a mean ADC value above 1313 × 10^−6^ mm^2^/s was associated with PsP (sensitivity = 98.3%; specificity = 100.0%). In a study of 35 patients, ADC maps were registered to contrast-enhanced T1-weighted images at baseline and follow up. Changes in relative ADC (rADC) values could differentiate PsP from TP when rADC decreased by 59.2% in patients with TP and by 18.6% in patients with PsP compared with baseline DWI, with a sensitivity and specificity of 86%, and an area under the curve (AUC) of 0.844 ± 0.065 (*p* = 0.014) [32]. The optimum decrease in the ADC ratio cut-off value for differentiating TP from PsP was 27.05% (sensitivity = 88%, specificity = 86%; *p* = 0.014). Prager et al. [33] demonstrated that the ADC decrease is greater in TP than in PsP. Table 1 sets out the results of these studies.

In another study, DTI revealed higher fractional anisotropy and reduced ADC values in the normal-appearing white matter adjacent to the edema in patients with RN, compared with patients with TP [34]. Nevertheless, neither DWI nor DTI provides sufficient information to accurately differentiate PsP from TP. Both yield heterogeneous signal intensities on DWI and ADC maps, with areas of reduced diffusion that may represent either highly cellular tumor areas or inflammatory processes [20].

#### 3.2.2. Perfusion-Weighted Imaging (PWI)

PWI is a set of imaging techniques for the study of blood flow and therefore requires an endogenous or exogenous tracer. Using gadolinium chelate as an exogenous contrast medium tracer is the most frequent way of performing perfusion measurements. After an intravenous injection of a gadolinium bolus [35], two basic techniques can be used. In dynamic contrast-enhanced MRI (DCE), T1-weighted sequences allow clinicians to assessing the increase in signal intensity due to gadolinium T1 effects. This technique is particularly suited for assessing contrast medium kinetics within tissue over a long time period and is hence mainly used in tumor assessment. The *K^trans^* is the marker obtained. With dynamic susceptibility contrast (DSC) MRI, dynamic T2*-weighted sequences are acquired before, during, and after contrast injection and used to evaluate regional brain perfusion parameters [35]. Owing to its T2* effects, gadolinium induces a signal loss over time and allows tissue microvascular density to be estimated through the measurement of cerebral blood volume (CBV) or cerebral blood flow [36]. With these techniques, the fact that the vessels after radiotherapy are modified with increased vascular permeability. Therefore, the *K^trans^* values and CBV values are more difficult to analyze than if the contrast agent stayed intravascular. Consequently, contradictory results in clinical studies on gliomas are frequent and depend on the vascular parameters in tumor of each patients. Arterial spin labeling (ASL) uses blood as an endogenous tracer [37]. The blood is labeled by an inversion or saturation pulse, leading to a change in signal intensity if it enters the slice(s) of interest DSC was found to be the most widely used technique for PWI in a study comparing all three perfusion methods [38,39].It has also been found to have the best diagnostic performance [40].

In three DSC studies, the mean relative CBV (rCBV) value was shown to be significantly lower in a radiation-induced brain injury group than in a glioma recurrence group [33,41,42]. However, these results are controversial [42,43]. Table 2 sets out the results of these studies. In a study of 39 patients, Thomas et al. [44] demonstrated in a DCE study that a *K^trans^* mean value of >3.6 had 69% sensitivity and 79% specificity for differentiating PsP from TP. Dynamic contrast-enhanced MRI yields lower *k^trans^* values in PsP than in TP [44,45], but these results are again controversial. In a prospective study, Yoo et al. demonstrated a significant difference in *K^trans^* values between patients with TP and PsP, with higher values for the latter [46]. ASL has been found to improve the diagnostic accuracy of DSC perfusion MRI in differentiating PsP from TP [47]. In a recent study, both 3D perfusion ASL and DSC perfusion MRI techniques had nearly equivalent performances for differentiating TP from PsP in patients with GBM. However, ASL seems to be less sensitive to susceptibility artifacts and may allow for improved classification in selected cases [48]. Gadolinium injection is required in GBM for a more accurate assessment. When it comes to comparing DWI and PWI, a meta-analysis included 24 studies on the differentiation of PsP from TP, a meta-analysis metaanalysis including 24 studies, with a total of 900 patients, found that DWI was slightly superior to PWI in terms of sensitivity (88% vs. 85%) and specificity (85% vs. 79%). When the authors compared the overall diagnostic accuracy of the MRI modalities, using their respective AUC values (0.9156 for DWI and 0.9072 for PWI), no significant difference emerged between the two [49].

#### 3.2.3. Spectroscopy

**Figure 3:**
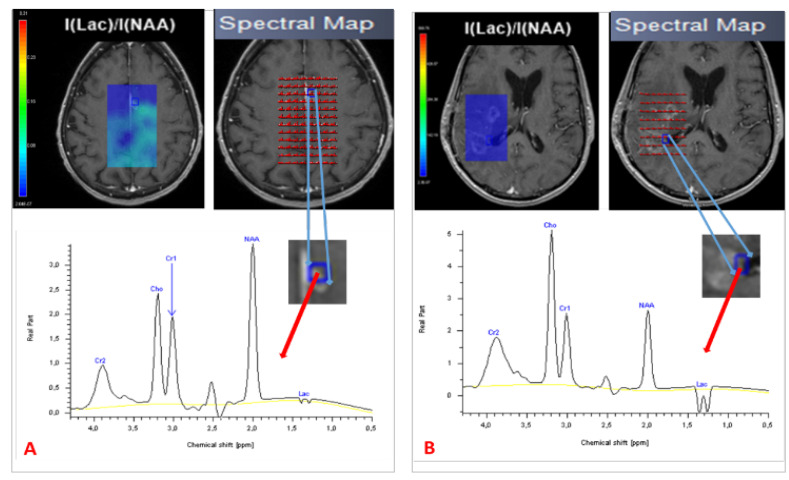
Spectroscopy of two patients treated for GBM, showing PsP with a normal spectrum (**A**) and TP with a high Cho/NAA ratio and Lac peak (**B**). TP = true progression: PsP = pseudoprogression; Cho = choline; NAA = *N*-acetyl aspartate; Lac = lactate.

Since the late 1980s, proton magnetic resonance spectroscopy (MRS) has been used to provide a noninvasive measure of brain metabolites [50]. Tumors have an abnormal metabolism compared with normal tissue. In that sense, MRS is valuable for establishing a clinical diagnosis, monitoring the effects of treatment and understanding disease mechanisms [51]. MRS is able to depict structural damage in brain tissue after RT before symptoms develop and before evidence of changes that can be observed using conventional MRI [20]. Different acquisition parameters can be modified to optimize MRS data acquisition. These parameters determine not only the appearance of the spectrum but also the information that can be extracted from it. One of the most relevant is echo time. At present, the echo time used in in vivo MRS by most groups ranges between 18 and 288 ms [52,53]. Three classes of spatial localization techniques are used in MRS: the single-voxel technique, which records spectra from one region of the brain at a time, and the multivoxel technique, in 2D, 3D, or whole-brain MRS, that records spectra from multiple regions and thereby maps the spatial distribution of metabolites within both normal tissue and most heterogeneous lesions dimensions [53]. Multivoxel spectroscopy is a chemical shift imaging of hydrogen and other atoms, such as phosphorus, sodium, and potassium, in different molecules, shown as a spectral pattern. MRI and MRS have provided important information on the pathophysiology of central nervous system radio-induced damage [54]. MRS has been investigated as a means of improving the detection of tumor infiltration [55]. It can detect and quantify different metabolites in vivo, with promising results for prognosis and treatment response [56,57] as well as for guiding radiotherapy dose painting [58]. Analysis of choline (Cho) and *N*-acetyl aspartate (NAA) peaks is of particular interest in studies of brain tumors [59]. Cho is a marker of cell membrane proliferation and is elevated in tumors. NAA is related to mature neuronal density and viability, and is decreased in tumoral tissue [60]. Lactate (Lac) is an end product of glycolysis and increases rapidly during hypoxia and ischemia. Creatine (Cr) peak is a marker for intracellular energy states that rarely varies and is used as an internal reference. A lipid peak appears in the event of membrane destruction due to necrosis [61]. Spectra from newly diagnosed or relapsing GBM differ from normal brain spectra, with decreased levels of NAA and often increased levels of Cho and Lac [53]. A Cho/NAA value >2 indicates a high-grade glioma [56]. Establishing a differential diagnosis between PsP and TP based on MRS findings is highly challenging, particularly with the use of single-voxel acquisitions. Both types of lesion can exhibit neuronal loss/dysfunction (low NAA), abnormal cellular membrane attenuation/integrity (high Cho), and anaerobic metabolism (high Lac/lipid ratio), (Figure 3). An elevated Cho/NAA ratio has been correlated with evidence of tumor recurrence [31,62,63,64]. To diagnose PsP, Sawlani et al. demonstrated elevated lipid signals on MRS [65]. An absence of Cho or a low Cho/NAA ratio was also observed. By contrast, patients with TP had lower lipid signals and a high Cho/NAA ratio. The presence of elevated lipid signals, along with a low Cho/NAA ratio, can help to differentiate PsP from TP [64]. Anbarloui et al. found that the mean Cho/NAA ratio for TP was 2.72, compared with just 1.46 for RN (*p* < 0.01) [66]. Another promising approach is 3D echoplanar spectroscopic imaging, which analyzes a large volume with greater resolution, as described by Verma et al., who distinguished TP from PsP in patients with GBM with a sensitivity of 94% and a specificity of 87% [67]. Table 3 sets out the results of these studies.

In a meta-analysis of 35 studies including 1174 patients treated for GBM, advanced MRI techniques were found to have greater diagnostic accuracy than conventional MRI for treatment response. PWI and MRS had the greatest sensitivity (91% and 92%) and specificity (95% and 85%) [22]. In a recent systematic review [68], Le Fèvre et al. proposed that in the case of suspected PsP after conventional MRI, DWI, and PWI should be performed first. Mean ADC value, ADC ratio, and mean rCBV would suggest TP if they had values <1.28–1.33, <1.40–1.55, and >1.82–3.01. If there was any remaining PsP doubt, MRS could then be performed to provide supplementary information. The proposed MRS ratios for differentiating between PsP and TP were Cho/NAA and Cho/Cr, with Cho/NAA < 1.47–2.11 and Cho/Cr < 0.82–2.25 indicating PsP. As a whole, all advanced MRI modalities seem to improve differentiation between PsP and TP, compared with conventional MRI. However, there are many limitations, and modalities need to be combined, with more in-depth use of the information they yield. Radiomics could be a powerful tool for performing this complex analysis.

#### 3.2.4. Radiomics and Pseudoprogression

**Figure 4:**
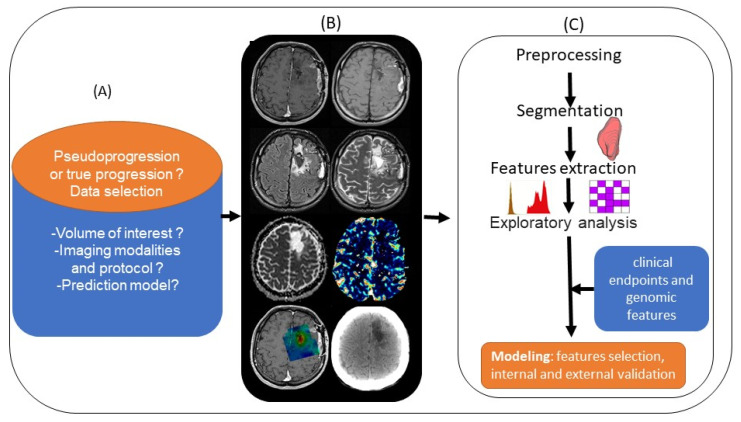
Standard pipeline of radiomics analysis applied to the differentiation of PsP from TP: (**A**) planning the radiomics study by asking basic questions. (**B**) Integrating multimodal images (left to right and top to bottom: T1 pre- and post-contrast enhancement, T2, FLAIR, ADC and rCBV map, metabolic MRSI map, and CT scan). (**C**) Preprocessing and segmentation of volume of interest in MRI images, with extraction of features from within the defined volume of interest quantifying tumor intensity, shape, and texture. After features selection, the radiomics features are combined with clinical and genomics data. A model is established after internal and external validation.

Medical imaging yields large amounts of information that are currently underused, and radiomics focuses precisely on ways of improving image analysis. It involves the high-throughput extraction of large numbers of image features and is one of the most recent innovations in medical imaging analysis [69]. The hypothesis is that the quantitative analysis of data for a given imaging modality using automated or semiautomated software can provide fuller and more complex information than a physician can. Tumors exhibit differences in shape and texture that can be measured using different imaging modalities [69]. Artificial intelligence approaches with radiomics cover the areas of diagnosis, prognosis, and treatment response. Improving disease stratification and advancing the personalized treatment of patients with glioblastoma appears to involve integrating radiomics into a multilayered decision framework with key molecular and clinical features [70]. Although this is an emerging topic, given the recency of the data and the different methodologies used by authors, radiomics analysis appears to involve the use of multimodal image acquisition (Figure 4A,B), segmentation or labeling, feature extraction, and finally statistical analysis (Figure 4C) to differentiate PsP from TP. Therefore, Chaddad et al. suggested that this means adopting optimized standard image processing, with a common criterion for performing segmentation, the fully automated extraction of radiomics features without redundancy, and robust statistical modeling validated in the prospective external setting [71]. Lambin et al. [72] recently proposed calculating a radiomics quality score to aid the assessment of both past and future radiomics studies.

#### 3.2.5. Differentiation between PsP and TP

Few studies have explored the potential of radiomics to differentiate between PsP and TP. Using T1, T2, and FLAIR, Bani et al. [73] assessed the value of radiomics features to diagnose PsP or TP in 76 patients (53 with TP and 23 with PsP). Patients were divided into training and validation groups in a 2:1 ratio, with survival balanced between the two groups. The authors found 11 features for classifying PsP and TP. Accuracy, sensitivity, and specificity were 75.0%, 81.6%, and 50.0% in the training set and 76.0%, 94.1%, and 37.5% in the validation set. When data on MGMT promotor methylation were included in this model, diagnostic performance improved, with accuracy of 83.0%, sensitivity of 88.9%, and specificity of 63.6% in the training set. During the validation phase, accuracy, sensitivity, and specificity were 79.2%, 80.0%, and 75.0% [73]. In another study of conventional MRI, Sun et al. [74] assessed the value of applying radiomics to T1-weighted contrast-enhanced imaging to differentiate between TP and PsP. The sample comprised 77 patients with GBM (51 with TP and 26 with PsP). The diagnostic efficacy of the radiomics classifier versus the assessments of three radiologists was further examined by considering accuracy, sensitivity, and specificity. The radiomics classifier was found to have accuracy, sensitivity, and specificity of 72.78%, 78.36%, and 61.33% versus 66.23% and 61.50% and 68.62%, 55.84%, 69.25%, and 49.13%, and 55.84%, 69.23%, and 47.06% for the radiologists.

In another study of 98 patients (76 with TP and 22 with PsP) using PWI [75], when *K^trans^* and rCBV were included in a radiomics model to distinguish between PsP and TP, accuracy reached 90.82% (AUC = 89.10%, sensitivity = 91.36%, and specificity = 88.24%; *p* = 0.017). The diagnostic performances of models built using the radiomics features from either *K^trans^* or rCBV were equally high (*K^trans^*: AUC = 94.69%, *p* = 0.012; rCBV: AUC = 89.8%, *p* = 0.004) [75]. In their conventional MRI study of 105 patients treated for GBM, including 59 in a training set (39 with TP and 21 with PsP) and 46 in a validation set (33 with TP and 13 with PsP), Ismail et al. [76] found that the two most discriminating features were local features, capturing the total curvature of the enhancing lesion with an accuracy of 84.75%, and the curvedness of the T2WI/FLAIR hyperintense perilesional region with an accuracy of 88.14% in the training set. In the validation set, accuracy was 83% for the enhancing lesion and 82.6% for the perilesional region. On both the enhancing lesion and perilesional region, accuracy was 91.5% for the training set and 90.2% for the validation set. Kim et al. [77] selected 12 significant radiomics features (three from conventional MRI, two from DWI, and seven from PWI) to construct their radiomics model in their study of 96 patients followed for GBM: a training set (26 with PsP and 35 with TP) and a validation set (20 with PsP and 14 with TP). In the training set, they demonstrated that the multiparametric (anatomical MRI, ADC, and CBV) radiomics model performed significantly better (AUC = 0.90, sensitivity = 91.4%, and specificity = 76.9%) than any single ADC (min and mean) or CBV (mean and maximum) parameter (AUC = 0.57–0.79, sensitivity = 0.629–0.771, specificity = 0.462–0.923; *p* < 0.05), and better than a monoparametric radiomics model using either conventional MRI (AUC = 0.76, sensitivity = 0.514, specificity = 0.885; *p* = 0.012), DWI (AUC = 0.78, sensitivity = 0.77, specificity = 0.76; *p* = 0.014), or PWI (AUC = 0.80, sensitivity = 0.657, specificity = 0.96; *p* = 0.43). In terms of external validation, the multiparametric model had a better diagnostic performance (AUC = 0.85, sensitivity = 0.714, specificity = 0.90) than any single approach, thus demonstrating its robustness. In a study of 35 patients, Baine et al. [78] found that a combination of two or three radiomics features was capable of predicting PsP on pre-RT MRI. Using radiomics in advanced MRI therefore seems to improve the identification of PsP, but it is too early to reach a formal conclusion, as more research is needed (Table 4). Multi-parametric MRI analysis using machine learning was used with success by several teams [79,80,81,82]. For example, Akbari et al. [80] described a signature based on multimodal MRI including DSC and DTI (but no MRS) with a high accuracy, keeping an accuracy of 75% in the interinstitutional validation cohort. In their conclusion, the authors outlined the possibility to integrate the proposed method into clinical studies via a freely available software.

These studies had several limitations. First, they each had a small patient sample, and all the patients were drawn from retrospective cohorts. With only a small number of patients, it seems quite difficult to reach any conclusion about radiomics. Second, different types of imaging protocol parameters (1.5T or 3T) were used for patient follow up [75,77]. Third, preprocessing methods and segmentation could be improved by using automatic techniques to limit intra- and interobserver variability. Fourth, biological data such as MGMT promoter and IDH status were not included in all the studies [54,73]. Fifth, no study evaluated spectroscopic imaging in a radiomics model, even though this has been shown to be a useful imaging technique for differentiating PsP from TP. Extracting information from these data is thus very challenging but seems to open up new perspectives for improving PsP diagnosis. To achieve a robust radiomics model, it would be useful to establish a rigorous evaluation criterion and follow recently published guidelines [15]. Using data from a prospective study with a large patient sample, including all advanced MRI imaging and biological data would improve the generalization of results. Moreover, imaging techniques are constantly evolving, and the spread of 3T magnets and the advent of ultra-high field MRI (7T) will improve the analysis of tumor metabolism and neuroinflammation. We can cite the use of phosphorous-31 spectroscopy, oxygen-17 imaging, carbon-13, and deuterium spectroscopy or ultrasmall superparamagnetic iron oxide (USPIO) contrast agents [83,84,85].

## 4. Conclusions

PsP occurs after GBM treatment, and its diagnosis remains a major challenge for radiologists and clinicians alike. MRI is the imaging modality of choice during follow-up. Nevertheless, neither conventional MRI nor advanced techniques can formally differentiate between PsP and TP. In routine clinical practice, follow-up MRI is most often used to distinguish between PsP and TP as surgery or biopsy for pathological confirmation is invasive and has its own drawbacks. For now, the RANO and modified RANO criteria combined with advanced MRI analysis are generally used in routine clinical practice and appear familiar to the medical community. Guidelines on the role of imaging in the management of progressive GMB in adults were recently updated by the Congress of Neurological Surgeon. These now suggest using MRI with and without gadolinium enhancement including DWI and MRS with a level II of evidence to differentiate TP from PsP. PWI is recommended with Level III evidence [15]. However, radiomics using conventional and advanced MRI techniques has demonstrated its ability to improve the accuracy of PsP diagnosis and could be a valuable tool in clinical practice. More studies are therefore needed. Large cohorts with multimodal follow up, particularly MRS imaging, and new emerging MR modalities would help to overcome this crucial diagnostic issue.

## Figures and Tables

**Table 1:** Review of DWI MRI studies. DWI = diffusion-weighted imaging, MRI = magnetic resonance imaging, N = number of patients, TP = true progression, PsP = pseudoprogression, ADC = apparent diffusion coefficient, rADC = relative apparent diffusion coefficient.

Study	*N*	Parameter	TP	PsP	*p*
Chu, 2013	30	5th percentile ADC 1000 5th percentile ADC 3000	906 × 10^−6^ mm^2^/s 587 × 10^−6^ mm^2^/s	1030 × 10^−6^ mm^2^/s 719 × 10^−6^ mm^2^/s	0.049 <0.001
Prager, 2015	68	ADC mean	1380 × 10^−6^ mm^2^/s	1590 × 10^−6^ mm^2^/s	0.003
Kazda, 2016	39	ADC mean	1155 × 10^−6^ mm^2^/s	1372 × 10^−6^ mm^2^/s	<0.001
Reimer, 2017	35	rADC decrease	59%	18%	0.005
Zhakari, 2018	17	ADC min in necrosis	1756 × 10^−6^ mm^2^/s	992 × 10^−6^ mm^2^/s	0.027

**Table 2:** Review of studies of DSC MRI. DSC = dynamic susceptibility contrast, MRI = magnetic resonance imaging, N = number of patients, TP = true progression, PsP = pseudoprogression, rCBV = relative cerebral blood volume.

Study	*N*	Parameter	TP	PsP	*p*
Young, 2013	20	rCBV mean	2.75	1.50	0.009
Prager, 2015	68	rCBV mean	1.81	1.015	0.003
Boxerman, 2017	19	rCBV mean	2.17	2.35	0.67
Wang, 2018	68	rCBV mean	3.39	1.39	<0.001
Rowe, 2018	67	Increase rCBV	73.7%	93.3%	-

**Table 3:** Review of studies of magnetic resonance spectroscopic imaging. *N* = number of patients, TP = True progression.

Study	*N*	Type of MRS	Parameter	TP	PsP	*p*
Smith, 2009	33	2D CSI	Median Cho/NAA	3.2	1.43	<0.001
			Median Cho/NAA	2.56	1.57	<0.001
			Median NAA/Cr	0.85	1.14	0.018
Elias, 2011	25	2D CSI	Mean Cho/NAA	2.81	1.39	0.0004
			Mean Cho/Cr	2.23	1.84	0.24
			Mean NAA/Cr	0.85	1.36	0.0033
Ambarloui, 2015	33	SV	Median Cho/NAA	2.72	1.46	0.01
			Median NAA/Cr	2.46	0.6	0.01
Bulik, 2015	24	2D CSI	Median CHO/NAA	2	0.77	<0.001
			Median Cho/Cr	0.45	0.99	<0.01
Kazda, 2016	39	2D CSI	Median Cho/NAA	2.13	0.74	<0.001
			Median Cho/Cr	0.89	0.64	0.013
			Median NAA/Cr	0.99	0.41	<0.001
Verma, 2018	27	3D EPSI	Cho/NAA	2.69	1.56	0.003
			Cho/Cr	1.74	1.34	0.023

PsP = pseudoprogression, Cho = choline, NAA = *N*-acetyl aspartate, Cr = creatinine, SV = single voxel, 2D CSI = two-dimensional chemical shift imaging, 3D EPSI = three-dimensional echo planar spectroscopic imaging.

**Table 4:** Studies that evaluated the use of radiomics models to differentiate between PsP and TP. Abbreviations: C: curvedness; Gd: gadolinium; *KT*: measure of total curvature; PsP: pseudoprogression; RANO: Response Assessment in Neuro-Oncology; S: sharpness; SI: shape index; SVM: support vector machine; TP: tumor progression; LOOCV: leave-one-out cross-validation; NA: not available; LASSO: least absolute shrinkage and selection operator; SMOTE: synthetic minority oversampling technique; SPCA: supervised principal component analysis; MMR = maximum relevance minimum redundancy. N = number of patients; Se: sensitivity; Sp: specificity. TP = true progression, PsP = pseudoprogression, DWI = diffusion-weighted imaging, DSC = dynamic susceptibility contrast, T1CE = T1 contrast-enhanced.

Study	Patients (*N*)	Imaging	Preprocessing	Segmentation	Feature Classification	Main Features or Parameters Found	Results	External Validation
Ismail, 2018	59:21 PsP and 38 TP	T1CE, T2, FLAIR	Skull stripping Intensity normalization	Manual	SVM 4-fold cross-validation	Mean of *KT* roundness, eccentricity Median of C, elongation shape factor	Accuracy: 91.5%	Yes Accuracy: 90.2%
Kim, 2019	61:26 PsP and 35 TP	T1CE, FLAIR, DSC DWI	hybrid white-stripe normalization excluding outliers inside the region of interes	Semi-automated	LASSO 10-fold cross-validation	14 features	Accuracy: 90% Se: 91.4% Sp: 76.9%	Yes Accuracy: 85% Se: 71.4% Sp: 90%
Elshafeey, 2019	98:76 TP and 22 PsP	T1CE, DSC	NA	Semi-automated	MMR SVM C5.0 LOOCV 10-fold cross-validation	*K^trans^* rCBV	Accuracy: 90.82% Se: 91.36% Sp: 88.2%	No
Bani-Sadr, 2019	76:53 TP and 23 PsP	FLAIR, T1CE	NA	Manual	SCPA 10-fold cross-validation	11 radiomic features	Accuracy: 75% Se: 81.6% Sp: 50%	Yes Accuracy: 76% Se: 94% Sp: 37.5%
Sun, 2021	77:51 TP and 26 PsP	T1CE	Normalization	Semi-automated	Random forest classification (SMOTE) 5-fold cross-validation	50 radiomic features	Accuracy: 72.78% Se: 78.36% Sp: 61.33%	No
Baine, 2021	35:27 TP and 8 PSP	T1CE	N4 Bias field correction Histogram matching normalization	Manual	ANOVA analysis 1000-time 3-fold cross-validations,	Wavelet_HHL_firstorder_Mean Original_firstorder_Minimum WaveLet LHL_glszm_SizeZoneNonUniformityNormalized	Mean AUC = 0.82 for the radiomic model	No
Akbari et al., 2020	63:35 TP, 10 Psp, 18 mixed response	T1CE, FLAIR, DSC DTI	Smoothed Correction of magnetic inhomogeneities Skull stripped	Manual	SVM LOOCV	1040 radiomics features analysed and 2 classifiers	Accuracy 87% to predict PSP, interinstitutional cohort accuracy 75%	yes
